# *Bartonella henselae* and *Bartonella quintana* antigens grown in liquid medium are inferior to cell culture-grown antigen for immunofluorescence IgG testing of patient sera

**DOI:** 10.1128/spectrum.01405-25

**Published:** 2025-07-25

**Authors:** Kristýna Dulavová, Iva Hammerbauerová, Kateřina Kybicová, Silke M. Besier, Agnes Hillebrecht, Heike Podlich, Mareike Steyer, Wibke Ballhorn, Volkhard A. J. Kempf

**Affiliations:** 1 Department of Parasitology, Faculty of Science, Charles University 37740, Prague, Czech Republic; 2 National Reference Laboratory for Lyme Borreliosis, Centre for Epidemiology and Microbiology, National Institute of Public Health, Prague, Czech Republic; 3 Institute for Medical Microbiology and Infection Control, University Hospital, Goethe University 14376, Frankfurt am Main, Germany; 4 National Reference Laboratory for Bartonella Infections, Frankfurt am Main, Germany; Inflammatix Inc., Sunnyvale, California, USA

**Keywords:** serodiagnostics, microscopy, evaluation

## Abstract

Serodiagnosis of *Bartonella* infections predominantly relies on immunofluorescence assays (IFA) utilizing antigen preparations derived from bacteria co-cultivated with host cells. We compared the diagnostic performances of whole-cell *Bartonella henselae* and *Bartonella quintana* antigens generated in liquid culture with those from cell culture. IFA using liquid culture-derived *B. henselae* antigens yielded lower IgG antibody titers, and no consistent correlation in IgG titers was observed for *B. quintana*. We conclude that liquid-cultured *Bartonella* antigens are inferior to those used in commercially available diagnostics.

**IMPORTANCE:**

In this study, we compare the diagnostic performances of *Bartonella henselae* and *Bartonella quintana* antigens cultivated in liquid medium with those of antigens derived from cell culture for use in IFA of patient sera. Our results demonstrate that liquid-cultured *Bartonella* antigens for IFA are less reliable and may lead to false or missed diagnoses. This highlights the importance of using validated, high-quality antigens to ensure accurate and trustworthy results in clinical practice.

## OBSERVATION

The spectrum of diseases caused by *Bartonella* spp. ranges from mild, localized infections to severe systemic illnesses. *Bartonella henselae* is the etiological agent of cat scratch disease. *Bartonella quintana* is responsible for trench fever (also known as Wolhynian fever or five-day fever), which today predominantly affects homeless people. In immunocompromised patients, both *B. henselae* and *B. quintana* are capable of inducing “culture-negative” endocarditis and vasoproliferative disorders, such as bacillary angiomatosis (1).

Serodiagnosis of *Bartonella* infections is primarily based on immunofluorescence assays (IFA) utilizing antigen preparations derived from bacteria co-cultivated with host cells (2, 3). These antigens are produced by inoculating host cells (e.g., Vero and HeLa cells) with *Bartonella* spp. previously cultured on blood-enriched agar plates (e.g., Columbia blood agar [CBA), followed by co-cultivation for up to 96 h. After fixation, the resulting antigen is employed in serodiagnostic testing, with threshold IgG titers considered diagnostic ideally ≥ 256 (≥320) to be evaluated positive (4). Currently, commercial serodiagnostic IFA kits are available for both *B. henselae* and *B. quintana* and offered by several manufacturers. Although enzyme-linked immunosorbent assay (ELISA)-based methods show promise, they remain less frequently utilized in routine diagnostics (5). Notably, the Centers for Disease Control and Prevention (CDC) do not recommend the use of agar-grown *Bartonella* as antigen sources in serodiagnostic procedures, as they do not represent the optimal antigen quality (4).

For several decades, the cultivation of *Bartonella* spp. has relied on enriched blood agar media. In 2008, we developed an easy-to-prepare *Bartonella* liquid (BaLi) medium supporting the planktonic growth of *Bartonella* spp. (6). This medium (based on Schneider’s *Drosophila* medium enriched with 10% heat-inactivated fetal calf serum and 5% sucrose) enables fast growth of various *Bartonella* species. Comparative analyses of *B. henselae* strain Marseille cultivated on CBA versus BaLi medium revealed no major differences in protein profiles, as assessed by Coomassie-stained SDS-PAGE and immunoblotting using rabbit immune serum or monoclonal antibodies. However, some variations in immunoreactivity were observed when patient sera were employed (6). To date, *Bartonella* antigen derived from BaLi culture has not been evaluated for its potential application in serodiagnostics.

In this study, we compared the diagnostic performances of *B. henselae* and *B. quintana* antigens cultivated in liquid medium with that of antigens derived from cell culture for use in IFA of patient sera. For this purpose, we analyzed 19 patient sera previously tested positive for anti-*B*. *henselae* and/or anti-*B*. *quintana* IgG, along with 20 control sera that had previously tested negative, using a commercially available immunofluorescence assay (Euroimmun, Lübeck, Germany) based on *B. henselae* Houston-I or *B. quintana* Fuller strains grown on HeLa cells. Immunofluorescence testing was done under strict quality-controlled criteria according to Deutsches Institut für Normung/International Organization for Standardization 15189:2014 standards (certificate number D-ML-13102-01-00). Ethical approval for this study was obtained from the local ethics committee (approval #423/11).

The following bacterial strains were used for the production of liquid culture-derived antigens: *B. henselae* Marseille (7), *B. henselae* Houston I variant I (8), and *B. quintana* JK31 (9). This was because of their known expression status of the trimeric autotransporter adhesins (*Bartonella* adhesin A [BadA]: *B. henselae* Houston I: BadA-negative, *B. henselae* Marseille: BadA-positive [10]; variably expressed outer membrane proteins [Vomps]: *B. quintana* JK 31: Vomp pos. [9]). Cultivation was performed in BaLi medium for 2 (*B. quintana*) or 3 (*B. henselae*) days until an optical density at 600 nm (OD₆₀₀) of approximately 0.5 was reached. Bacteria were harvested by centrifugation at 4,991 × *g* for 20 min at 4°C and subsequently washed with Dulbecco’s phosphate-buffered saline (DPBS; Gibco, Thermo Scientific, Rockford, USA). Bacteria were fixed in 2% paraformaldehyde (PFA; Sigma-Aldrich, Taufkirchen, Germany) and spotted onto 24-well microscope slides (StarFrost 24-well, Knittel Glas, Braunschweig, Germany). After air-drying, slides were washed three times with 1× DPBS (5 min each), followed by overnight blocking at 4°C with 0.2% bovine serum albumin (BSA; Sigma-Aldrich) dissolved in DPBS.

Human sera from patients with PCR-confirmed or clinically highly suspected *Bartonella* infections were obtained from the serum repository of the National Reference Laboratory for *Bartonella*, Frankfurt, Germany. Sera were serially diluted in 1× DPBS, starting at a dilution of 1:80 and extending up to 1:81,920. As positive controls, rabbit antisera against *B. henselae* Marseille (11), *B. henselae* Houston I variant I (10), or *B. quintana* JK31 (prepared analogously to the method described by Kaiser et al. [11]; data not shown) were used.

The incubation of the antigens with sera and secondary antibodies was performed for 1 h at room temperature, followed by three washes with 1× DPBS/0.2% Tween-20. Positive (rabbit sera, diluted 1:800) and negative controls (omitting rabbit or human sera) were included in all staining procedures (data not shown). The following secondary antibodies were used: Alexa 488-conjugated donkey anti-human F(ab′)₂ fragment IgG (H + L; 1:400) and Alexa 488-conjugated goat anti-rabbit IgG (H + L; 1:200), both from Jackson ImmunoResearch, Cambridgeshire, UK. Bacterial DNA was stained with 4′,6-diamidino-2-phenylindole (DAPI; 2 µg/mL; Merck, Darmstadt, Germany) for 10 min at 4°C. Slides were mounted using a fluorescence mounting medium (Dako, Hamburg, Germany) and analyzed using a Zeiss Axio Imager 2 microscope equipped with a Spot RT3 camera (Diagnostic Instruments, Inc., MI, USA) operated via VisiView V.2.0.5 software (Visitron Systems, Puchheim, Germany).

**Fig 1:**
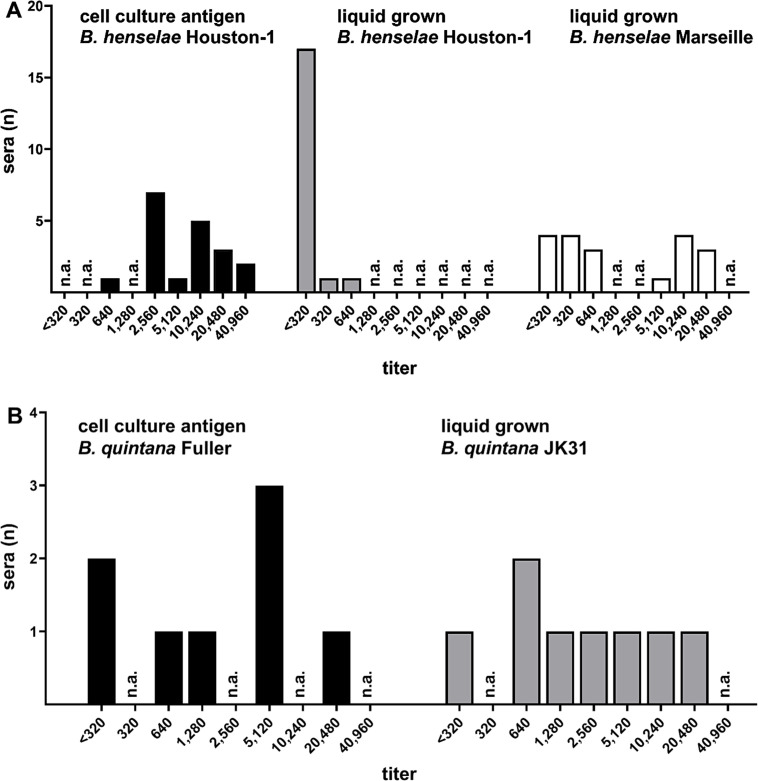
Comparison of the antigen reactivities between liquid-grown *B. henselae* and *B. quintana* antigens and commercially available cell-culture antigens. The number of reactive sera is shown on the *y*-axis, and the titers are shown on the *x*-axis. (**A**) Reactive sera (*n* = 19) tested with *B. henselae* antigens (cell-culture-grown *B. henselae* Houston I, liquid-grown *B. henselae* Houston I, or *B. henselae* Marseille). (**B**) Patient sera (*n* = 8) PCR-positive for *B. quintana* infection, reactive with *B. quintana* antigens (cell-culture-derived: *B. quintana* fuller, liquid-grown *B. quintana* JK31).

Sera were classified as “positive” at a titer of ≥320 and “negative” at a titer of <320 for both commercial (cell culture)- and liquid (BaLi)-grown antigens. The evaluation of *B. henselae* IgG antibodies yielded the following results (Fig. 1A): when *B. henselae* Houston-1 bacteria grown in BaLi medium were used as antigen, 17 out of 19 sera previously tested positive appeared negative; two sera, which had reacted at a titer of 10,240 with the commercial antigen, showed reactivity at a titer of 320 or 640, resulting in a loss of reactivity by four or five titer steps, respectively; and all sera that tested negative with the cell-culture antigen also tested negative with the BaLi-grown *B. henselae* Houston I antigen.

When *B. henselae* Marseille bacteria grown in BaLi-medium were used as antigen, four of the 19 sera that tested positive in the commercial IFA (all at a titer of 2,560) were negative with the *B. henselae* Marseille antigen. Three sera showed identical results for both antigens (two with a titer of 10,240 and one with a titer of 640). Ten sera exhibited lower titers with the BaLi-grown *B. henselae* Marseille antigen (onefold reduction: *n* = 4, twofold reduction: *n* = 1, threefold reduction: *n* = 3, sixfold reduction: *n* = 1, sevenfold reduction: *n* = 1). Two sera showed slightly higher titers with the BaLi-grown antigen (commercial IFT titer: 10,240, BaLi-grown titer: 20,480) compared to the cell culture-derived antigen. Additionally, five of the 20 sera that tested negative in the commercial IFT were positive when tested with BaLi-grown antigen (all at a titer of 320).

Eight serum samples from patients with PCR-confirmed *Bartonella* infections (detected by specific PCRs [12; data not shown]) were used to evaluate *B. quintana* IgG antibodies (Fig. 1B). Two of these sera, which had previously tested negative in the commercial IFA, showed positive titers of 640 and 2,560 with the BaLi-grown antigen. One serum that tested positive in the commercial IFA (titer 5,120) was negative with the BaLi-grown antigen. Two sera that had previously tested positive in the commercial IFA demonstrated reduced reactivity with the BaLi-grown antigen (titers reduced from 5,120 to 1,280, from 1,280 to 640, and from 20,480 to 2,560 corresponding to a loss of one to three titer steps). All sera that tested negative with the cell-culture antigen were also negative with the BaLi-grown *B. quintana* JK31 antigen.

In our diagnostic study, we utilized the best-characterized human sera available, including those with clinical diagnoses of *Bartonella* infections, positive serology using commercial antigens, and in some cases, even PCR-confirmed infections. Despite this, discrepancies in seroreactivity were observed between commercial cell culture- and BaLi-grown antigens. The underlying reasons for these differences remain unclear.

When evaluating human sera with the BaLi-grown antigen, fluorescence signals were only partially visible on the bacterial surface, often appearing as round, nonspecific signals rather than clearly delineating bacterial morphology. In contrast, technical controls using specific rabbit antisera (see above) revealed strong staining of all bacteria across all three antigen preparations (data not shown). This may be explained by the fact that the rabbit antisera exhibit high reactivity due to repetitive immunization with inactivated bacteria expressing a defined protein pattern, thereby inducing a robust immune response directed against a set of antigens and proteins.

In contrast, for human sera, neither the time point of infection nor the infecting bacterial strain is known. This alone can be expected to lead to a considerable variability in the specific immune response, a phenomenon that has been previously described (13). Moreover, it cannot be excluded that the protein expression profile of the bacterial strains used in the commercial IFA differs from that of the strains used in this study. For instance, “phase variation” due to the loss of expression of immunodominant outer membrane proteins (e.g., *Bartonella* adhesin A) during cultivation has been reported (14).

Taken together, our findings demonstrate that IFA testing utilizing liquid culture-derived *B. henselae* antigens generally results in lower or even undetectable antibody titers compared to those obtained with commercially available cell culture-derived antigens. For serum samples from patients infected with *B. quintana*, no clear correlation of the IgG titers was observed. While efforts to simplify the preparation of IFA antigens are understandable, we must conclude that liquid-cultured *Bartonella* antigens are inferior to those used in commercially available diagnostics.

## Data Availability

All data are provided within the article.
